# A bacterial tungsten-containing aldehyde oxidoreductase forms an enzymatic decorated protein nanowire

**DOI:** 10.1126/sciadv.adg6689

**Published:** 2023-06-02

**Authors:** Agnieszka Winiarska, Fidel Ramírez-Amador, Dominik Hege, Yvonne Gemmecker, Simone Prinz, Georg Hochberg, Johann Heider, Maciej Szaleniec, Jan Michael Schuller

**Affiliations:** ^1^ Jerzy Haber Institute of Catalysis and Surface Chemistry Polish Academy of Sciences, Kraków, Poland.; ^2^ SYNMIKRO Research Center and Department of Chemistry, Philipps-University of Marburg, Marburg, Germany.; ^3^ Faculty of Biology, Philipps-University of Marburg, Marburg, Germany.; ^4^ Max Planck Institute of Biophysics, Frankfurt am Main, Germany.; ^5^ Max Planck Institute for Terrestrial Microbiology, Marburg, Germany.

## Abstract

Aldehyde oxidoreductases (AORs) are tungsten enzymes catalyzing the oxidation of many different aldehydes to the corresponding carboxylic acids. In contrast to other known AORs, the enzyme from the denitrifying betaproteobacterium *Aromatoleum aromaticum* (AOR_Aa_) consists of three different subunits (AorABC) and uses nicotinamide adenine dinucleotide (NAD) as an electron acceptor. Here, we reveal that the enzyme forms filaments of repeating AorAB protomers that are capped by a single NAD-binding AorC subunit, based on solving its structure via cryo–electron microscopy. The polyferredoxin-like subunit AorA oligomerizes to an electron-conducting nanowire that is decorated with enzymatically active and W-cofactor (W-co) containing AorB subunits. Our structure further reveals the binding mode of the native substrate benzoate in the AorB active site. This, together with quantum mechanics:molecular mechanics (QM:MM)–based modeling for the coordination of the W-co, enables formulation of a hypothetical catalytic mechanism that paves the way to further engineering for applications in synthetic biology and biotechnology.

## Teaser

The formation of electron conducting nanowire filaments explain the superior properties of a bacterial aldehyde oxidoreductase.

## INTRODUCTION

Many bacteria and archaea expand the catalytic repertoire of their enzymatic reactions by using either molybdenum (Mo) or tungsten (W) as catalytic transition metals. Molybdo- and tungstoenzymes act as dehydrogenases, oxidases, hydroxylases, hydratases, or reductases, catalyzing key steps of metabolism, many of which are fundamentally important for global nutrient cycles and bioremediation. W-containing enzymes are classified into two families, namely, the dimethyl sulfoxide (DMSO) reductase family, which contains mostly molybdoenzymes, and the aldehyde oxidoreductase (AOR) family, which consists almost exclusively of tungstoenzymes. A hallmark feature of the DMSO reductase family is that the metal is bound to the dithiolene groups of two metallopterin (MPT) guanine dinucleotide cofactors and an amino acid (Cys, Sec, Asp, or Ser) as an additional ligand, whereas the AOR family contains almost exclusively W enzymes with two bound MPT cofactors per W, but no ligand from the protein (*1*–*3*).

The AOR enzymes are catalyzing the oxidation of many different aldehydes to corresponding carboxylic acids with ferredoxins or viologen dyes as electron acceptors (*4*). Consequently, the primary physiological role of AOR enzymes appears to be detoxification of surplus and cytotoxic aldehydes. These enzymes are the only known biocatalysts that can also catalyze the thermodynamically difficult reverse reaction, the reduction of nonactivated carboxylic acids to aldehydes at *E*′ ≈ −560 mV (*1*), if low-potential electron donors are available (*5*, *6*). This reductive potential of AORs has attracted increased attention, specifically for developing alcohol-producing variants of syngas-fermenting acetogenic bacteria (*7*).

Although they can perform such highly sophisticated redox reactions, our structural and mechanistic understanding of AOR enzymes has been sparse. Now, the only structurally characterized W AORs come from the hyperthermophilic archaeon *Pyrococcus furiosus*: namely, AOR (sensu stricto) (*8*), formaldehyde oxidoreductase (FOR) (*9*), and W-containing oxidoreductase 5 (WOR5) (*10*). Those structures revealed the fold of the AOR catalytic subunit and general structure of the MPT and W cofactor. However, the details on W coordination were inconclusive because of the low local resolution and intrinsic spectroscopic properties of the metal atoms (*1*, *8*, *11*).

In mesophilic bacteria, however, AORs often occur as multiprotein complexes in which they interact with other subunits that likely enable alternative electron acceptors to ferredoxin (*12*–*14*). No structural information or mechanistic understanding of the molecular architecture and function of these multisubunit AORs is yet available. Recently, the multisubunit AOR from the denitrifying betaproteobacterium *Aromatoleum aromaticum* was characterized (AOR_Aa_) (*12*). The enzyme is involved in the anaerobic degradation of phenylalanine, benzyl amine, and benzyl alcohol, not only mainly detoxifying surplus aldehyde intermediates but also taking over large-scale oxidation of these aldehydes in the absence of dedicated dehydrogenases (*15*–*17*). In addition to the catalytic W-containing subunit AorB and the FeS cluster containing protein AorA, the protein contains an additional flavin adenine dinucleotide (FAD)–containing subunit (AorC) that enables the enzyme to reduce nicotinamide adenine dinucleotide (NAD^+^) alternatively to viologen dyes (*12*). This composition differs substantially from that of the other studied AOR family members (*1*). The enzyme was initially suggested to be a heterohexamer of the three subunits with the W cofactor linked to a FAD cofactor by a chain of five Fe_4_S_4_ clusters, which suits the observed use of either NAD^+^ or benzyl viologen (BV^2+^) as alternative redox mediators. AOR_Aa_ has a broad substrate spectrum, specifically oxidizing aromatic, heterocyclic, aliphatic, and halogenated aldehydes. The reduction of carboxylic acids is catalyzed by AOR_Aa_ in the presence of either artificial low-potential electron donors or hydrogen, showing a substrate spectrum of the reverse reaction comparable with that of aldehyde oxidation (*12*). The unprecedented ability of this enzyme to use hydrogen as an electron donor for acid or NAD^+^ reduction qualifies the enzyme as a new type of hydrogenase (*18*). In addition, the enzyme can also be coupled to an electrochemical cell (*19*). AOR_Aa_ is insensitive to oxygen in cell extracts, stable in isolated form and active at room temperature. These properties together with a wide substrate range, as well as its ability to use easily available electron mediators for aldehyde oxidation (e.g., NAD^+^, viologens, or other dyes), make the enzyme a prime candidate for applications in synthetic biology and biotechnology.

Here, we show the molecular structure of AOR_Aa_, the first bacterial multisubunit AOR, with a global resolution of 3.22 Å. Unexpectedly, our structure reveals that an extended protein filament is formed by AorAB protomers that is nucleated by a single FAD-containing AorC subunit. The repeating units are connected through a polyferredoxin chain generated by lined-up ferredoxin-like AorA subunits, essentially forming an electron-conducting “nanowire” decorated with enzymatically active W-cofactor (W-co) containing AorB subunits, reminiscent of another recently characterized polymer-forming redox enzyme, the hydrogen-dependent CO_2_ reductase (HDCR) (*20*). This form of redox enzyme has the potential to stabilize the complex and to link enzymatic subunits through the filament to increase their catalytic activity.

## RESULTS

### AOR forms electron-conducting filaments

AOR from *A. aromaticum* was produced by heterologous expression in *Aromatoleum evansii* with an added twin-Strep-tag at the N terminus of the small subunit AorA to facilitate the purification of the complex by affinity chromatography on a streptavidin column (*18*). Minor impurities in the obtained preparation were removed by a subsequent run of the enzyme over a size exclusion column. The purified AOR_Aa_ catalyzed the oxidation of 1 mM benzaldehyde with NAD^+^ as an electron acceptor with a specific activity of 18 U mg^−1^.

The enzyme eluted from the size exclusion column in an unexpectedly broad peak with a mean retention time corresponding to a mass of 300 kDa (fig. S1A), suggesting that the enzyme does not form a homogeneous quaternary structure. This has been confirmed by mass photometry (MP) analysis of the preparation. The mass distribution histograms indicate the presence of protein species of 80, 133, 221, 306, and 392 kDa (fig. S1C). These masses correspond to sole AorAB protomers (86 kDa), a heterotrimeric type of complex (AorABC; 132 kDa), and three larger complexes containing subsequently more AorAB protomers: Aor(AB)_2_C (218 kDa), Aor(AB)_3_C (304 kDa), and Aor(AB)_4_C (392 kDa). In accordance with the previous data on AOR_Aa_ (*21*), the five-subunit Aor(AB)_2_C complex appears to be the most abundant in the preparation studied. Therefore, AOR_Aa_ apparently forms oligomeric structures of Aor(AB)*_n_*C composition. We observed that the complexes tend to dissociate when diluted, suggesting an even higher potential polymerization grade under undisturbed in vivo conditions.

**Fig. 1.**
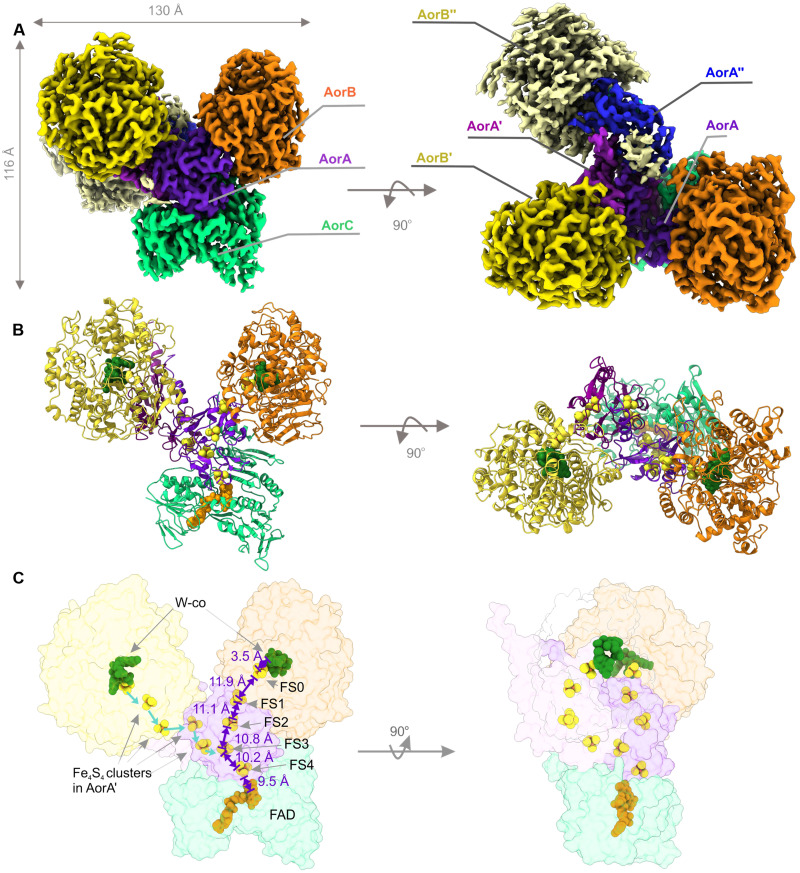
Overall structure of AOR_Aa_ complex. (**A**) Representative three-dimensional cryo-EM reconstructed density of AOR (up) colored by identified subunits. The low-resolution density was assigned to another (third) AorA″ and AorB″ subunit. (**B**) Three-dimensional reconstruction of AOR_Aa_ consisting of two protomers: AorABC and AorA'B'. (**C**) The electron transfer pathway of AOR_Aa_. Both acid reduction and hydrogen oxidation hypothetically can occur on one of the two W-cofactors (W-co; W-*bis*-MPT). The chain of Fe_4_S_4_ clusters transfers the electrons from the W-co to the FAD, where NAD^+^ is reduced. The electron transfer chain of the distal protomer (blue) is linked to the main electron pathway (violet).

We subjected the purified AOR_Aa_ complex to cryo–electron microscopy (cryo-EM) analysis, yielding a reconstruction of a 3.22-Å density map of seven subunits of an Aor(AB)_3_C complex (Fig. 1 and fig. S2). Unexpectedly, the AOR_Aa_ complex forms a short filament. Our reconstruction allowed the model building of an Aor(AB)_2_C subcomplex with good geometry and refinement statistics, while the third AorAB protomer of the complex showed too low resolution for full structural assessment. The side chain densities of the core residues of the protein are clearly visible, and densities of all cofactors appear intact (fig. S3).

The filaments originate from a single AorC subunit and polymerize to short oligomeric chains of added AorAB protomers. The filamentous form of the complex is characterized by an observed helical twist of 110° and 24-Å rise (translation) per AB dimer along the filament axis. Although we do not know the actual oligomerization grade in vivo, we observed the presence of two to four AorAB protomers experimentally with purified protein and can confidently assume even higher oligomerization grades in vivo, judging from the large amounts of free AorAB protomers in preparation, which probably arise from dissociation of larger complexes during the purification process.

### Filamentation is mediated by the C-terminal helices of AorA

AorA was identified as the central element of the oligomeric complex. The protein shows a typical double-ferredoxin fold. It binds four iron-sulfur clusters through conserved [CxxCxxCxxxC] motifs, which we numbered FS1 to FS4. FS1 and FS2 mediate the electronic connectivity to the AorB subunits (11.9 Å from FS1 to the FeS cluster FS0 in AorB), and FS4 of the proximal AorA subunits connects to the FAD cofactor in AorC (9.5 Å from FS4, shown in Fig. 1C). The clusters form a clear pathway, allowing electron flow between the W cofactors and the bound FAD nucleotide in AorC. FS3 and FS4 form a spiral core that traverses the length of the filament and are likely responsible for electronic connectivity between the protomers. The short distances below 15 Å between all redox cofactors are expected to support rapid electron transfer via quantum tunneling within microseconds. The presence of AorA or similar FeS cluster–containing subunits has only been observed in a few clades of the AOR family, such as the AOR_Aa_, bacterial glyceraldehyde-3-phosphate oxidoreducase (GOR), WOR5, and Bam clades, while the other characterized members (including AOR_Pf_ and FOR) do not contain such a subunit (*1*, *10*, *22*). Moreover, none of the other known W enzymes with such a subunit form oligomers.

**Fig. 2.**
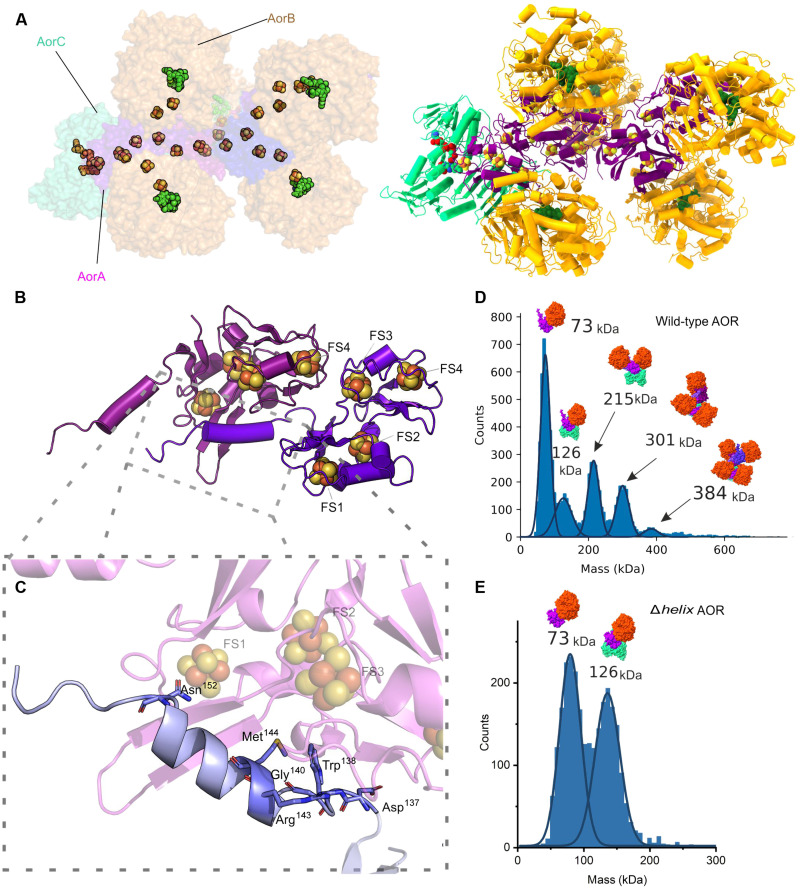
Filament formation in the AOR_Aa_ complex. (**A**) Modeled AOR complex of composition Aor(AB)_5_C. (**B**) Cartoon model of AorAA′ with (**C**) a close-up view with atomic depiction of residues at the C-terminal helix of AorA responsible for binding of both subunits; in the helix mutant, the last amino acid in the C terminus of AorA is D137. (**D**) Mass histogram of the undissociated AOR_Aa_ and (**E**) helix mutant (both of 50 nM concentration) sample fitted with distribution peaks of Gaussian function. The average molecular mass of the distribution (kDa) shown along with a scheme of corresponding complex composition.

A unique feature of AorA is the structure of the C-terminal 20 amino acids, which are highly conserved in the sequences of the AOR_Aa_ subclade members but not in any other related sequence. These residues form an α helix protruding from the ferredoxin fold, which resembles similar structures known from filament-forming redox proteins, such as HDCR (*20*). This C-terminal helix of AorA provides an additional surface to extend the interface with the next AorA subunit in the oligomer, probably facilitating filament formation by hydrogen bridges and hydrophobic interactions (Fig. 2).

To prove that the C-terminal fragment of AorA has a major role in filament formation, we constructed a variant protein Δ*helix*-AOR lacking the AorA residues from 138 to the C terminus. The purified Δ*helix*-AOR exhibited twofold lower specific activity than wild-type AOR, either for benzaldehyde oxidation with BV^2+^ as an electron acceptor or for NAD^+^ reduction with hydrogen as an electron donor. Unexpectedly, in this variant, we did not observe a change of the specific activity of hydrogen-dependent acid reduction (table S2). The stoichiometry of the complex was measured using mass photometry. Here, the mass histograms of Δ*helix*-AOR (Fig. 2E) show only two macromolecular complexes, corresponding to the AorAB protomer and an AorABC complex. No further peaks corresponding to higher-organized structures were seen. Therefore, we demonstrated that the deletion of the C-terminal helix of AorA abolishes filament formation, further validating our structural observations.

### The FAD-containing subunit AorC allows the filament nucleation

**Fig. 3.**
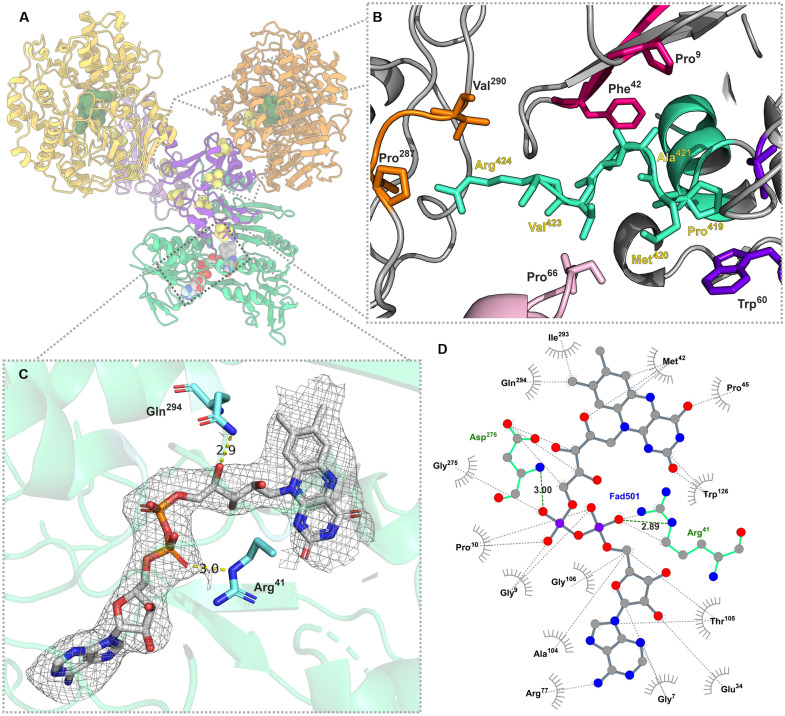
Structural properties of the FAD-containing subunit. AorC shown (**A**) in the Aor(AB)_2_C complex, and in structural details, (**B**) C-terminal helix of AorC (depicted in cyan). Atoms show amino acids responsible for interactions with other subunits. R424 of AorC forms a hydrogen bond with AorB (P287) and has hydrophobic interactions with V290 of AorB. M420 of AorC interacts with AorA′ by weak hydrophobic interactions (F42 and P9 residues in AorA′). Lastly, M420 and P419 in the AorC helix interact even with AorA″ (W60 and A95). (**C**) FAD cofactor in the corresponding electron density and depiction of main nucleotide binding interactions with the protein by Gln^294^ and Arg^41^; (**D**) ligand-protein interaction map for the FAD cofactor.

The AorC subunit features the nucleation site of the complex, as shown by the many interactions that are formed between AorC and most of the other subunits of the Aor(AB)_3_C complex. AorC contains a characteristic Rossmann fold of six parallel β strands interspersed by α helices (*23*) that is conserved in FAD-binding structures, with an extended C-terminal domain of three α helices. This domain might have an important task in the nucleation of the complex, as it takes part in binding not only all three subsequent AorA subunits in the Aor(AB)_3_C complex but also AorB through a salt bridge (Fig. 3B).

The largest interface in the complex (1662 Å^2^) is formed between AorC and the proximal AorA, which takes up to 16% of the surface of the latter. However, AorC also interacts with AorB by forming a smaller interface of 434 Å^2^. The extent of interactions may explain the relative stability of the basic AorABC complex, as it was observed in mass photometry. However, AorC does act as a binding interface not only for the first AorAB protomer but also for the second and third AorAB protomers of the complex. The respective electron transfer subunits AorA′ and AorA″ of the subsequent protomers together occupy a less-important contact interface of 474 Å^2^.

The FAD cofactor was well resolved in the electron density, and the two main motifs binding the FAD in the protein can be identified. The conserved Arg^41^ bonds with the 2′-phosphate by a salt bond, while Glu^294^ interacts via an H-bond with one of the ribitol hydroxyl groups of FAD (Fig. 3D). Met^42^ is probably involved during electron transfer, as it is in close contact with FAD and two of the cysteines that coordinate the FS4 cluster in AorA. The isoalloxazine ring of the FAD cofactor is accessible for NAD-type substrates via a characteristic binding pocket of FAD-dependent oxidoreductases. There, a highly conserved Arg^179^ residue is placed at the entrance to the pocket, providing a binding domain for the phosphates of the NAD^+^ cofactor (fig. S7).

### AorB binds a W cofactor

**Fig. 4.**
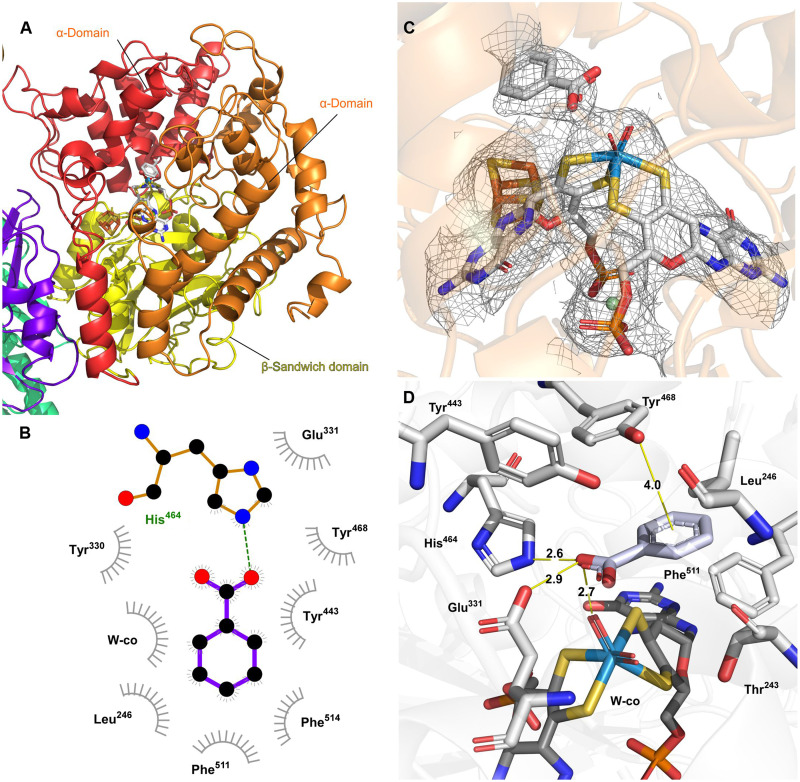
Structural properties of the catalytic subunit. (**A**) AorB secondary structure with two α-domains shown in red and orange and a β-sandwich domain colored yellow. (**B**) Ligand-protein interaction map for benzoic acid present in density. (**C**) W-co and Fe_4_S_4_ cofactors and benzoic acid in the corresponding density. (**D**) Anticipated interactions of benzoic acid with residues in the active site.

The AorB subunits of the AOR_Aa_ oligomer show structural homology to both the AOR (50% sequence identity) and the FOR (30% sequence identity) subunits from *P. furiosus* (figs. S5 and S6), reflecting a close relation between these W AOR enzymes. The W cofactor is placed in a binding pocket formed between the three domains of AorB, in a similar fashion to AOR and FOR. A β-sandwich domain (residues 1 to 211) forms a base that is linked to the W-*bis*-MPT (W-co) cofactor by a magnesium ion bridging the MPT phosphates, as well as two α domains (residues 212 to 418 and 419 to 616), both surrounding one of the pterin moieties of the two MPT residues (Fig. 4). The magnesium ion present in W-co is coordinated to their phosphate groups and links the cofactor to the protein through coordination with the backbone carbonyl oxygens of the highly conserved Asn^92^ and Ala^182^ residues. The density corresponding to the W cofactor shows the expected pyranopterin form for both MPT, which are bound to the protein by hydrogen bonds with residues Arg^75^, Gly^94^, Arg^181^, Asp^361^, Asp^504^, Ile^508^, Cys^509^, and Val^510^. These residues are conserved and appear similarly used as in AOR_Pf,_ whereas they are not conserved in FOR, which exhibits some changes in the W-co binding motifs. No further metal ions were identified in close proximity to any of the pterins in AOR_Aa_, in contrast to the available structures AOR_Pf_, FOR_Pf_, and WOR5 that show sodium (*8*), calcium (*9*), or two additional magnesium ions (*10*), respectively.

The highly conserved residues His^484^ and Glu^331^ (79 and 82% conserved throughout the AOR family, respectively) are located close to the W and therefore are possible candidates for being catalytically active residues. We suggest that they provide binding contacts and proton relay sites for the substrates, i.e., either a (hydrated) aldehyde or a carboxy group.

One additional Fe_4_S_4_ cluster (named FS0; Fig. 1C) was identified within van der Waals distance to the W cofactor (3.5 Å edge to edge). In AOR_Aa_, this Fe_4_S_4_ cluster is bound by four cysteines (Cys^295^, Cys^298^, Cys^302^, and Cys^509^), and the same conserved motif of cysteines is present in all known structures and virtually all protein sequences of the AOR family members (99% of proteins). In contrast to the structure of AOR_Pf_, we do not observe bridging of the W cofactor and Fe_4_S_4_ cluster via a conserved arginine side chain (Arg^75^ in Aor_Aa_), which instead interacts through hydrogen bonding with a phosphate oxygen of one of the MPT cofactors. Moreover, the proximal MPT forms a direct H-bond with the Sγ atom of Cys^509^, one of the ligands of FS0.

The electron density map allowed the assessment of the W-*bis*-MPT cofactors in the AorB subunits, including tracing of the phosphate residues with the coordinating magnesium and determining the geometry of the ligands coordinating the W atom as distorted octahedral. The dithiolenes of the two MPT cofactors were verified as W ligands, and an excessive electron density surrounding the W atom indicated the presence of two additional small ligands; although, the obtained resolution was not sufficient to clearly identify them. Overall, the W-*bis*-MPT cofactor density exhibited a similar geometry to the previously reported x-ray structure of AOR_Pf_ (*R*^2^ = 0.963326) (*8*). Unfortunately, the existing structural assignment of AOR_Pf_ does not provide any clues on the ligand geometry of the W center, because it was refined almost 30 years ago without any other known Mo or W enzyme structures as references and shows no small ligands around the W atom (*8*, *24*). Therefore, we further refined the AOR_Pf_ structure from the original electron density map using theoretical modeling (QM:MM). Initial inspection of the electron density suggested the presence of two potential ligands of the W atom. Consequently, we have modeled active site W-co versions with different sets of bound ligands (figs. S8 and S9) using the AOR_Pf_ structure as a structural template. The resulting optimized six-coordinate cofactor structures were fitted into the models of the cryo-EM structure of AOR_Aa_ (Fig. 4C). The best candidate structure for the density available corresponds to the oxidized version of cofactor [W(VI)] containing two oxo ligands (fig. S10). On the basis of the observed distorted octahedral cofactor geometry and the known differences in pterin geometries between oxidized and reduced cofactor models (*25*, *26*), we infer an oxidized state of the W-co in the available density maps. The presence of two oxo ligands is also in good agreement with the available spectroscopic data for AOR_Pf_ (*27*, *28*). One of the oxo ligands of AOR_Aa_ may interact via H-bonding with His^464^, which could be in a double-protonated form as it occurs in the ethylbenzene dehydrogenase from the DMSO reductase family (*29*). Furthermore, the gamma-carboxy group of Glu^331^, which penetrates the active site cavity, may also assist in acid-base catalysis.

### W-co directly interacts with the substrate in AorB

Two large, hydrophobic channels exhibiting neutral charge on their surfaces lead directly to the coordinating sphere of the W atom, allowing the access of larger molecules (fig. S4). No protein ligands were identified directly coordinating the W. Nevertheless, a clearly visible cofactor-bound substrate containing an aromatic ring is clearly visible, which is assumed to be benzoate carried over from the growth medium. One of the oxygen atoms of the substrate is bound to the cofactor by hydrogen bonding at 2.7 Å (as shown in Fig. 4D). The substrate is kept in place by hydrophobic interactions with Phe^511^ and Phe^514^, as well as π-π stacking with Tyr^468^. All these aromatic residues are conserved in AOR_Pf_ and most enzymes of the more closely related subclades within the AOR family, but not in the enzymes of the FOR, archaeal glyceraldehyde-3-phosphate oxidoreducase (GAPOR), or GOR subclades, which do not convert aromatic substrates. In the case of WOR5, both Phe^511^ and Tyr^468^ are conserved, consistent with its substrate range containing aromatic aldehydes. Thus, our structure explains ligand binding modes in AOR and related enzymes.

## DISCUSSION

We present here a cryo-EM structure of AOR_Aa_ as a heteromultimeric W-dependent AOR, whose active-site AorB subunits are closely related to those of previously characterized members of the AOR enzyme family and contain a W-*bis*-MPT cofactor and a Fe_4_S_4_ cluster (FS0). Unexpectedly, the enzyme forms enzymatic filaments, shielding its Fe_4_S_4_ clusters from surface exposure while providing the possibility of “supercharging” the complex with many electrons, which rationalizes some of the biotechnologically favorable characteristics of AOR_Aa_, such as its very low oxygen sensitivity, high stability, and high catalytic rate, as well as the previously observed burst kinetic behavior of hydrogen-dependent acid reduction (*12*, *21*).

The described assembly of AOR_Aa_ as a multimeric, filamentous form is an emerging way to influence the activity of metabolic enzymes. There is a newfound understanding that the quaternary structure greatly affects enzymatic activity (*30*). For example, the acetyl-CoA (acetyl coenzyme A) carboxylase from different higher eukaryotes and yeast has been shown to form higher-order structures as a mechanism for enzyme activity regulation. The polymerized enzyme exhibited higher activity than the monomeric fraction of the enzyme (*31*, *32*). A similar phenomenon has been observed for the bifunctional CoA-dependent acetaldehyde and alcohol dehydrogenase AdhE of *Escherichia coli* (*30*, *33*) and the HDCR, a key enzyme of the acetogenic metabolism of *Acetobacterium woodii* (*30*) and *Thermoanaerobacter kivui* (*20*).

The effect of filamentation in HDCR was recently extensively studied and proven to affect enzyme activity in a differential manner by increasing formate production dependent on the filament length. Similar to AOR_Aa_, filamentation of HDCR is mediated by the C-terminal helices of (in this case, two) polyferredoxin subunits HycB3 and HycB4, which was proven by site-directed mutagenesis in both enzymes. Similar to AorA of AOR_Aa_, these two subunits of HDCR form the core of a nanofilament and bind enzymatically active counterparts (*20*). However, neither HycB3 nor HycB4 are orthologous to AorA, although they present a similar overall structure: All three subunits consist of two fused bacterial ferredoxin domains, which superimpose well structurally (root mean square deviation of 3.62; fig. S11). Compared to AorA, the C-terminal helices of the two subunits HycB3 and HycB4 are longer and have a more defined secondary structure.

Inspired by these similarities, we modeled higher-order oligomers of Aor(AB)*_n_*C to prove that the interface geometry allows for the binding of more than the four protomers that we observed in mass photometry (fig. S11). While the higher-order structure of larger complexes might have been lost during purification, we presume that the complexes with two or three AorAB protomers might be stabilized by surface contacts of these protomers with AorC. Thus, the AorC subunit might be the key factor for the formation of higher-order complexes by providing a stable rump oligomer, which would be further elongated with more weakly bound AorAB protomers without direct contact to AorC. The existence of long filaments is possible in vivo, but can only be established or ruled out by means of tomography or other in vivo methods.

The electron transfer link between the Fe_4_S_4_ clusters of subsequent AorA subunits may even directly connect different catalytic AorB subunits along the wire, allowing electron flow within the filamentous structure. For example, it is possible that one AorB subunit in the oligomer oxidizes hydrogen (or an aldehyde) at the same time while another one reduces an acid molecule. Such a phenomenon could affect the flexibility of the enzyme reactions, as well as the enzyme kinetics. Possible direct electron links between two W cofactors may enable cooperativity of the active sites, and binding of a substrate in the active site of one subunit could influence the affinity or reactivity of other active sites.

**Fig. 5.**
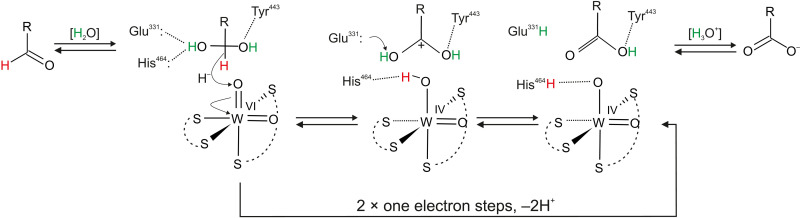
Proposed mechanism of aldehyde oxidation by AOR_Aa_. Aldehyde undergoes spontaneous addition of water to form respective geminal diol. Deprotonated Glu^331^ and His^464^ and protonated Tyr^443^ bind the geminal diol in a position where the α-hydrogen forms a hydrogen bond with oxo ligand coordinating W-co. This hydride is abstracted and the proton from the hydroxy group is intercepted by the Glu^331^, and acid is formed.

On the basis of the structural insight provided by cryo-EM of the active site and its ligand binding mode as well as results from QM:MM modeling of the W-*bis*-MPT cofactor, we propose a mechanistic hypothesis for the reaction mechanism of AOR (Fig. 5). Aldehydes in aqueous solutions exist in equilibrium with 1,1-geminal diols at the carbonyl atom (e.g., 99% of formaldehyde is hydrated) (*34*). We assume that this activated form of the aldehyde has the most beneficial geometry to bind to the active site, because it is similar to the produced carboxylic acid. Our product-bound structure suggests that the catalytic mechanism occurs in the second ligand sphere of the W-co, as found in many recent studies of other Mo or W enzyme mechanisms (*22*). Thus, we propose that one of the hydroxyl groups of a bound hydrated aldehyde forms H-bonds with His^464^ and Glu^331^. At the same time, the other OH group may receive an H-bond from Tyr^443^ (part of a proton relay system reaching the surface). This arrangement directs the carbonyl H atom of the hydrated aldehyde toward one of the oxo ligands of W-co (approximately 2.3 Å). The H-bonds formed with the basic residues activate the substrate by electron donation, supporting a formal hydride shift, which yields a reduced W-co [W(IV)O(OH)(MPT)_2_]. The transiently formed carbocation intermediate of the aldehyde would immediately transfer a proton to Glu^331^ (or His^464^), forming the carboxylic acid. The catalytically active oxidized form of the W-co would be restored via two one-electron transfers through the chain of FeS clusters toward the FAD, as well as the transfer of two protons from W-co and Glu^331^ to the solvent via the proton relay system. In that way, the active form of the cofactor is restored, while the produced acid may be released from the active site either before or after reoxidation of the W-co. The proposed mechanism differs from a previously proposed mechanism on the basis of the structure of FOR (*35*) but agrees with previous studies on the other AOR family members while also providing a plausible mechanism for the reverse reaction of acid reduction (*27*). Therefore, despite awaiting experimental validation, we suggest this as a general feature for AOR reactivity (*8*).

Our structural understanding of the molecular architecture of this enzymatic, decorated electron nanowire and the mechanistic insights we obtained about its active site architecture and catalytic cycle pave the way to further engineering of AOR for applications in synthetic biology and biotechnology. Examples are the production of aromatic or long-chain aliphatic alcohols from acids as potential biofuels (*21*), as well as the conversion of acids to other useful products by coupling AOR-dependent aldehyde production to other aldehyde-converting enzymes, e.g., for forming Schiff bases or C─C condensations. It is even conceivable that W enzymes new to nature can be generated on the basis of the AOR scaffold by using the nanowire backbone and exchanging the AorB subunits with those providing different enzymatic activities.

## METHODS

### Cell growth

*A. evansii* containing the AOR expression plasmid [as described by Winiarska *et al.* (*21*); carrying genes *aorA*: WP_011238651, *aorB*: WP_011238652, *aorC*: WP_011238653, *aorD*: WP_041646405, and *aorE*: WP_041646407] was grown anaerobically in a minimal medium using benzoate as the sole carbon source and nitrate as the electron acceptor as described previously (*21*). Cultivation was carried out in an anaerobic 30-liter fermenter (30°C, 7 days) with periodic supplementation of nitrates and sodium benzoate (to a concentration of 10 and 4 mM, respectively), monitoring nitrate and nitrite levels, with the addition of ampicillin and Na_2_WO_4_ to a final concentration of 100 μg/ml and 18 nM, respectively. Cultivation was continued until the OD_578_ (optical density at 578 nm) was 0.6, the temperature was lowered to 18°C, and the enzyme overexpression was induced by the addition of anhydrotetracycline to a concentration in the culture medium of 200 ng/ml. The culture was resupplemented with Na_2_WO_4_ to a final concentration of 10 μM. After 20 hours of incubation, cells were harvested from the culture medium by centrifugation at 4500*g* (1 hour, 4°C).

### Purification of AOR

The cell suspension was lysed by sonication, and cell debris was separated by ultracentrifugation at 40,000*g* for 1 hour at 4°C. The cell-free extract was then applied to a Strep-tag II column (5 ml; IBA GmbH), and after rinsing with a stock buffer [100 mM tris-HCl (pH 8.0), 150 mM NaCl, and 10% (w/v) glycerol], the enzyme was eluted with a buffer containing stock buffer enriched with 10 mM desthiobiotin. The collected fractions were then concentrated to 3 ml by ultrafiltration (Centricon) using a 30-kDa cutoff and applied on gel filtration using a 120-ml Superdex 200 column (16/600) equilibrated with buffer [100 mM Hepes (pH 7.5), 150 mM NaCl, and 10% glycerol]. AOR fractions were stored anaerobically at −80°C until further use without evident loss of AOR activity.

The purification of mutant Δ*helix*-AOR was conducted as above, but without the step on the Superdex 200 column. Activities of both preparations were measured spectrophotometrically, as shown previously (*18*).

### Cryo-EM protein sample preparation

The preparation of solution for application on EM grids from the stock of frozen protein was carried out under anaerobic conditions. The frozen stocks containing AOR_Aa_ (4.6 mg/ml) in 100 mM Hepes (pH 7.5), 150 mM NaCl, and 10% (v/v) glycerol were thawed on ice. The samples were prepared by diluting the protein stock with 45 mM Hepes (pH 7.5) and 50 mM NaCl to a final concentration of 0.7 mg/ml of AOR, 50 mM Hepes, and glycerol below 2% (v/v).

### Preparation of cross-linked samples

The samples prepared with additional cross-linking with bis(sulfosuccinimidyl)suberate (BS3; Thermo Fisher Scientific) were prepared under the same conditions as native samples. The additional step of cross-linking was optimized by conducting the reaction with BS3 according to the manufacturer’s instructions (adding 0.5, 1, 1.5, or 3 mM BS3). The general procedure for cross-linking was started with the preparation of a fresh 10 mM stock solution of BS3 in water and then preparing the cross-linking mixtures with the respective concentration of BS3. The cross-linking reaction was started with the addition of 8.7 μl of AOR to the final concentration of 1 mg/ml; the total volume of each reaction was 40 μl. The reaction was incubated for 30 min at 25°C and subsequently quenched by adding 1 M tris (pH 7.5) to a final concentration of 30 mM. The quenching reaction was incubated for 15 min at 25°C. The sample for cryo-EM analysis was prepared with 1 mM BS3 and diluted to achieve a concentration of 0.7 mg/ml of protein.

### Preparation of EM grids

Quantifoil R 2/1 on 200 copper mesh (Quantifoil Micro Tools GmbH) was used immediately after glow discharging in a glow discharge cleaning system (PELCO easiGlow) for 25 s with 15-mA current to make the grid more hydrophilic. A series of grids containing AOR samples were prepared by vitrification using an automatic plunge freezing device (Vitrobot Mark IV). Four microliters of sample was manually placed on a grid, followed by automatic blotting of the sample with filter paper to remove excessive moisture and plunging the grid into liquid ethane.

### Data collection and processing

Data collection was conducted by the technical support staff at the Department of Structural Biology, Central Electron Microscopy Facility, Max Planck Institute of Biophysics on a Glacios cryo-TEM (Thermo Fisher Scientific). The microscope was working with an accelerating voltage of 200 kV. The camera Falcon 3 in electron counting mode was operating with a raw pixel size of 1 Å, total exposure dose of 40 e^−^/Å^2^, and spherical aberration of 2.7 mm. For the resolved sample, 896 micrographs were processed with cryoSPARC software (*36*).

The images were corrected for the estimated beam-induced motion and contrast transfer function. Initially, particles were picked with a blob picker and extracted from a subset with the best 811 micrographs. A reference-free two-dimensional (2D) classification resulted in 549,381 particles that were further used to optimize the parameters in the template pick of only 100 micrographs. Three rounds of reference-free 2D classification, Topaz training, and particle extraction were performed to constrain the particle candidates, resulting in 192,383 particles after parameters were applied to the entire dataset. Particles were then subjected to ab initio reconstruction deriving in three independent classes from which the best two were refined using the nonuniform refinement algorithm. A final step of per-particle defocus and global contrast transfer function (CTF) parameter optimization led to a final calculated resolution of 3.22 Å for the AOR complex.

### Model building

The reconstruction of the protein chain was facilitated by the construction of a homology model in AlphaFold (*37*) of each subunit, which was then fitted in Chimera (*38*) for electron density. The model was fitted into the reconstructed density using COOT (*39*) and iteratively refined using Phenix real-space refinement (*36*). Images of density and built model were prepared using UCSF Chimera (*38*), UCSF ChimeraX (*40*), and PyMOL (*41*). The model was validated on the basis of the Ramachandran plot. More than 98% of the residues should have a main chain conformation consistent with the Ramachandran distribution, and any outliers should be justified by the strained surrounding (cofactors) and density map.

### Mass photometry

The measurements were performed using a OneMP mass photometer (Refeyn Ltd., Oxford, UK), while data acquisition were performed using AcquireMP (Refeyn Ltd. v2.3). MP movies were recorded at 1 kHz, with exposure times varying between 0.6 and 0.9 ms, adjusted to maximize camera counts while avoiding saturation. Microscope slides (70 mm by 26 mm) were cleaned for 5 min in 50% (v/v) isopropanol (HPLC grade in Milli-Q H_2_O) and pure Milli-Q H_2_O, followed by drying with a pressurized air stream. Silicon gaskets to hold the sample drops were cleaned in the same manner and fixed to clean glass slides immediately before measurement. The instrument was calibrated using the NativeMark Protein Standard (Thermo Fisher Scientific) immediately before measurements. Each protein was measured in a new gasket well (i.e., each well was used once). To find focus, 18 μl of fresh buffer [10 mM tris (pH 8.0), 100 mM KCl, and 0.1 mM EDTA] adjusted to room temperature was pipetted into a well, and the focal position was identified and locked using the autofocus function of the instrument. The measurement was started right after the addition of 2 μl of sample to the well, resulting in a 10-fold dilution of the sample just before measurement. Each sample was measured in three repetitions. The data analysis was conducted using DiscoverMP software, which converts the contrast values to molecular masses based on calibration.

### Mutagenesis

For the production of mutant Δ*helix*-AOR, the expression plasmid was amplified by inverse polymerase chain reaction using 5′-phosphorylated primers (forward: 5′-TGAGAGGAGCGGACATCATGGGATGGAATCG-3′, reverse: 5′-CGTCCAGTCGGCATCGATGTAGGTGATCG-3′). The resulting DNA was circularized in a blunt end ligation reaction, resulting in a modified version of the plasmid with a shortened *aorA* gene that lacks the 57 base pairs before the stop codon.

### QM:MM modeling

The geometry of the W cofactor in AOR_Aa_ was obtained on the basis of a reinterpretation of the crystal structure of AOR_Pf_ [Protein Data Bank (PDB) code 1AOR]. In the 1AOR structure, the model of the W cofactor lacks the ligands of the W atom, resulting in excess electron density. On the basis of available knowledge of W- and Mo-containing enzymes as well as results of extended x-ray absorption fine structure data collected for AOR_Pf_, different models of the active site were constructed. First, a model with W(VI) and two oxo ligands was constructed, from which models with W(IV), one hydroxo and one oxo ligand or one oxo and one water ligand, were derived. The W(VI)OO model charge was neutralized with 13 sodium ions, after which it was soaked with TIP3P solvent molecules (as water models) and subjected to geometry minimization using the AMBER package with ff03 force field (*42*, *43*). The missing parameters for the AOR cofactor were obtained according to an established protocol (*44*) at the B3LYP level of theory in the gas phase with Gaussian16 with a 6-31g(d,p) basis set for light atoms, as well as 6-311g(d) for S and LANL2DZ basis set and pseudopotential for W with solvent correction calculated according to integral equation formalism variant of the Polarizable Continuum Model (ε = 4.0, radius of solvent 1.4). Point charges were computed using Merz-Kollman electron density calculations (*45*) in Gaussian16, followed by the Restrained Electrostatic Potential (RESP) procedure available in the Antechamber of the AmberTools (see the Supplementary Materials) (*43*). After minimization, the protein with a 5-Å shell of water molecules was cut out and subjected to a two-step geometry minimization by the QM:MM method. The model contained 15,870 atoms and was partitioned into two parts for QM and MM modeling, respectively. The QM region contained the whole W(VI) atom coordinated by two oxo ligands and two pterins in dihydro form, and the Mg^2+^ ion was coordinated by two H_2_O ligands and phosphate groups from the pterins. The cofactor, all residues, and solvent molecules in a 12-Å radius around the W atom were subjected to geometry minimization, while the coordinates of the rest of the model were frozen. In the first step, the geometry was minimized using a mechanical embedding scheme and, subsequently, with an electronic embedding scheme. The reduced models of the W(IV)-containing cofactor were obtained by replacement of the oxo ligands at W atom with OH or OH_2_ groups, respectively, and geometry optimization was performed with just mechanical embedding [for W(IV)OOH_2_] or with additional electronic embedding [for W(VI)OO and W(IV)O(OH)]. The obtained geometries were used to fit the electron density of the AOR_Aa_ model.

## Notes

View/request a protocol for this paper from *Bio-protocol*.
