# Supplementary material for: A bacterial tungsten-containing aldehyde oxidoreductase forms an enzymatic decorated protein nanowire
Source: Sci Adv. 2023 Jun 2;9(22):eadg6689. doi: 10.1126/sciadv.adg6689 (PMC10413684; doi:10.1126/sciadv.adg6689)
Supplement: Supplementary file 1 — Figs. S1 to S11 Tables S1 and S2 Legends for data S1 to S9 [file sciadv.adg6689_sm.pdf]

Science Advances
AAAS

Supplementary Materials for

A bacterial tungsten-containing aldehyde oxidoreductase forms an enzymatic decorated protein nanowire

Agnieszka Winiarska *et al.*

Corresponding author: Jan Michael Schuller, jan.schuller@synmikro.uni-marburg.de;
Johann Heider, heider@staff.uni-marburg.de; Maciej Szaleniec, maciej.szaleniec@ikifp.edu.pl;
Agnieszka Winiarska, agnieszka.winiarska@ikifp.edu.pl

Sci. Adv. **9**, eadg6689 (2023)
DOI: 10.1126/sciadv.adg6689

The PDF file includes:

Figs. S1 to S11
Tables S1 and S2
Legends for data S1 to S9

Other Supplementary Material for this manuscript includes the following:

Data S1 to S9

Supplementary Figures and Tables

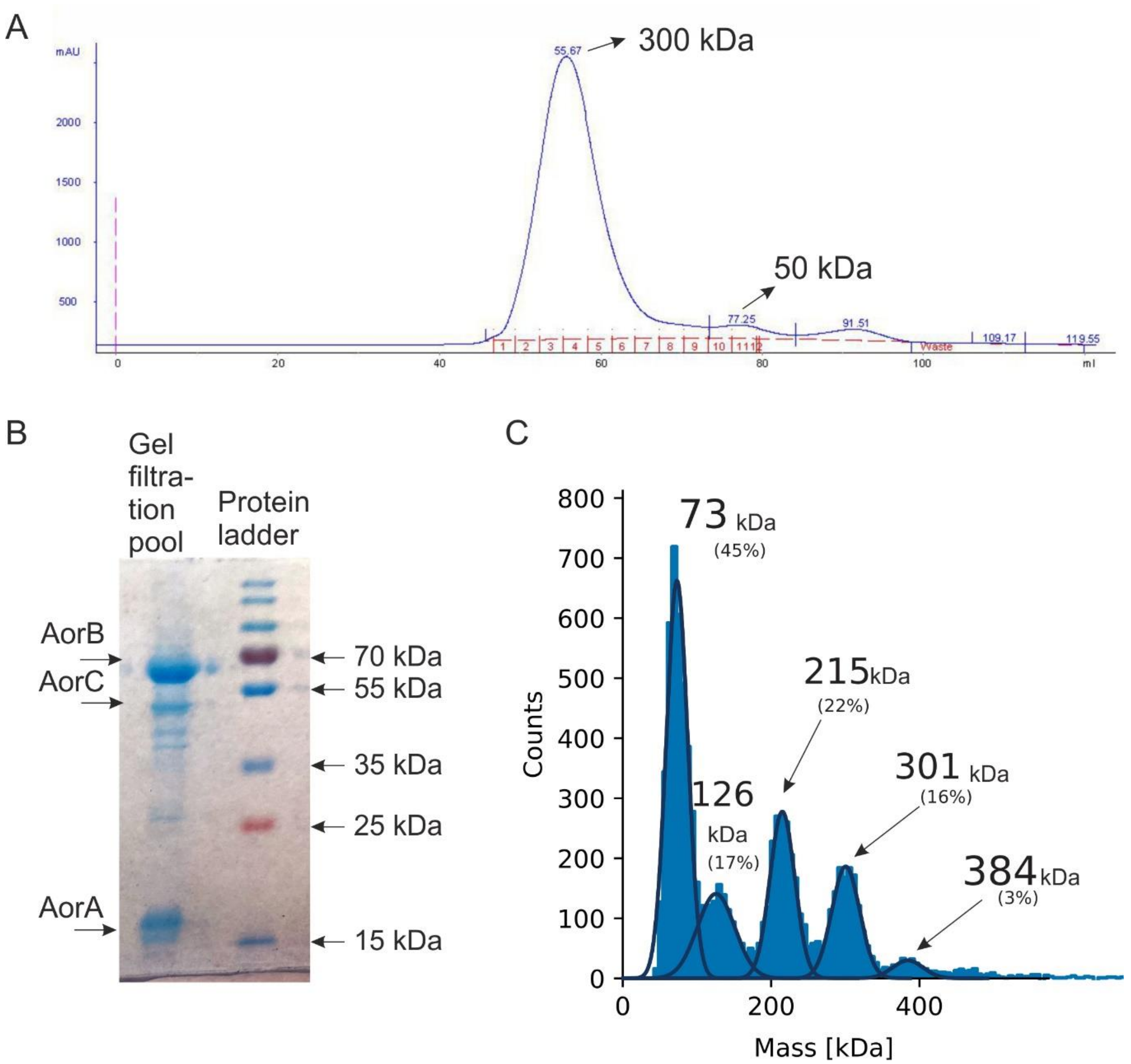

Fig. S1. Characterization of AOR_{Aa} preparation purity and complex stoichiometry. A) Chromatogram obtained by size exclusion chromatography where AOR_{Aa} elutes as a major peak corresponding with a molecular weight of 300 kDa, according to an external calibration curve; B) Protein separation on SDS-PAGE of the AOR_{Aa} pool from the size exclusion chromatography in A); C) Mass histogram of the undissociated AOR_{Aa} (50 nM).

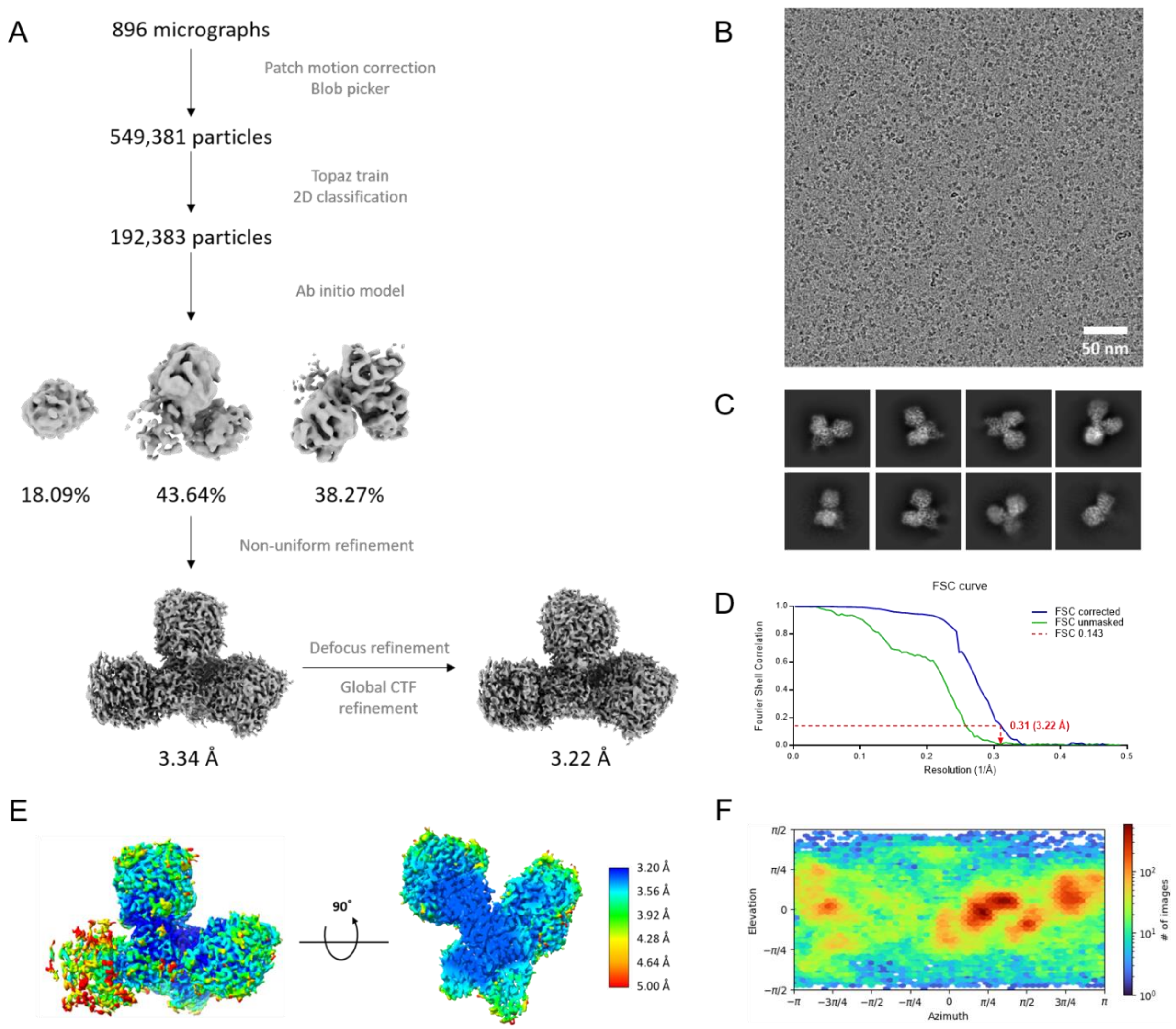

Fig. S2 Data processing. A) Overview of the cryo-EM data-processing scheme; B) Representative cryo-EM micrograph collected on a Glacios Cryo-TEM operated at 200 kV and equipped with a Falcon 3 camera; C) Reference-free 2D class averages showing AOR in multiple orientations. The edge of each box corresponds to 256 Å; D) Fourier Shell Correlation (FSC) curves of unmasked and corrected 3D maps; E) Density map of AOR_{Aa} coloured according to the local resolution calculated by cryoSPARC (left) and cut-open view of the central section tilted by 90° (right); F) Angular distribution of the particles used for the final round of refinement.

Table S1. Cryo-EM data collection, refinement and validation statistics.

Protein	AOR_{Aa}
EMDB ID – $Aor(AB)_3C$	EMD-16376
PDB ID – $Aor(AB)_2C$	8C0Z
**Data collection and processing**	
Microscope	Glacios Cryo-TEM
Voltage (kV)	200
Camera	Falcon 3
Total electron exposure ($e^-/Å^2$)	40
Defocus range (μm)	-0.8 to -3.0
Software	cryoSPARC
Raw pixel size (Å)	1.00
Symmetry imposed	C1
Micrographs (no.)	896
Initial extracted particles (no.)	549,381
Final extracted particles (no.)	79,731
Final map resolution (Å)	3.22
FSC threshold	0.143
Map sharpening B-factor ($Å^2$)	137.2
**Refinement**	
Initial model used	AlphaFold
Model composition	
Chains	5
Non-hydrogen atoms	15,083
Protein residues	1972
Ligands	2 x Benzoate, 2 x Mg^{2+}, 2 x W-co, 1 x FAD, 10 x 4Fe-4S
B factors	
Protein	126.86
Ligand	64.52
R.M.S Deviations	
Bond lengths (Å)	0.004
Bond angles (°)	0.674
**Validation**	
MolProbity score	1.46
Clash score	2.69
Poor rotamers (%)	0.13
Ramachandran plot	
Favoured (%)	93.93
Allowed (%)	5.91
Outliers (%)	0.15

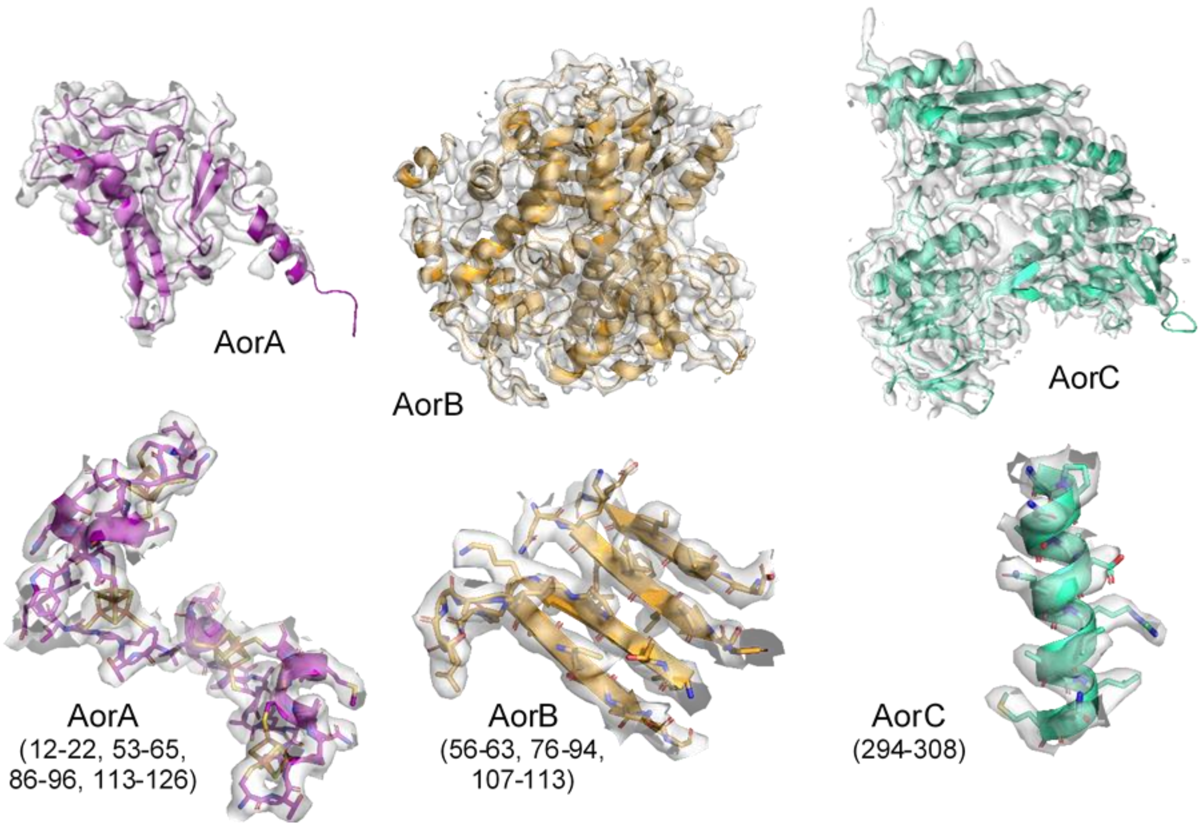

Fig. S3. Representative regions of each subunit in their electron density map. Representative regions of the AorA, AorB, and AorC subunits with their surrounding electron density maps. Maps are displayed as a surface using a contour level of up to 2 Å around the atoms. The colour code matches Fig. 1.

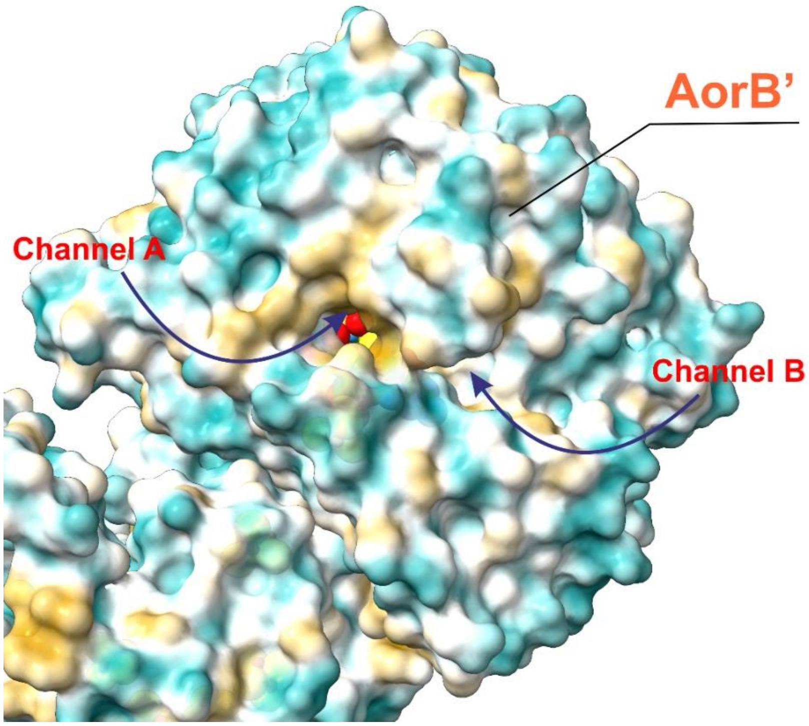

Fig. S4. Channels leading to W-co in the AorB' subunit. The surface is coloured according to its residue hydrophobicity (cyan: less hydrophobic; orange: more hydrophobic). The entrance to active site is partially covered by a helix formed by i.a. by active site residues Val477, Pro478 and Phe514.

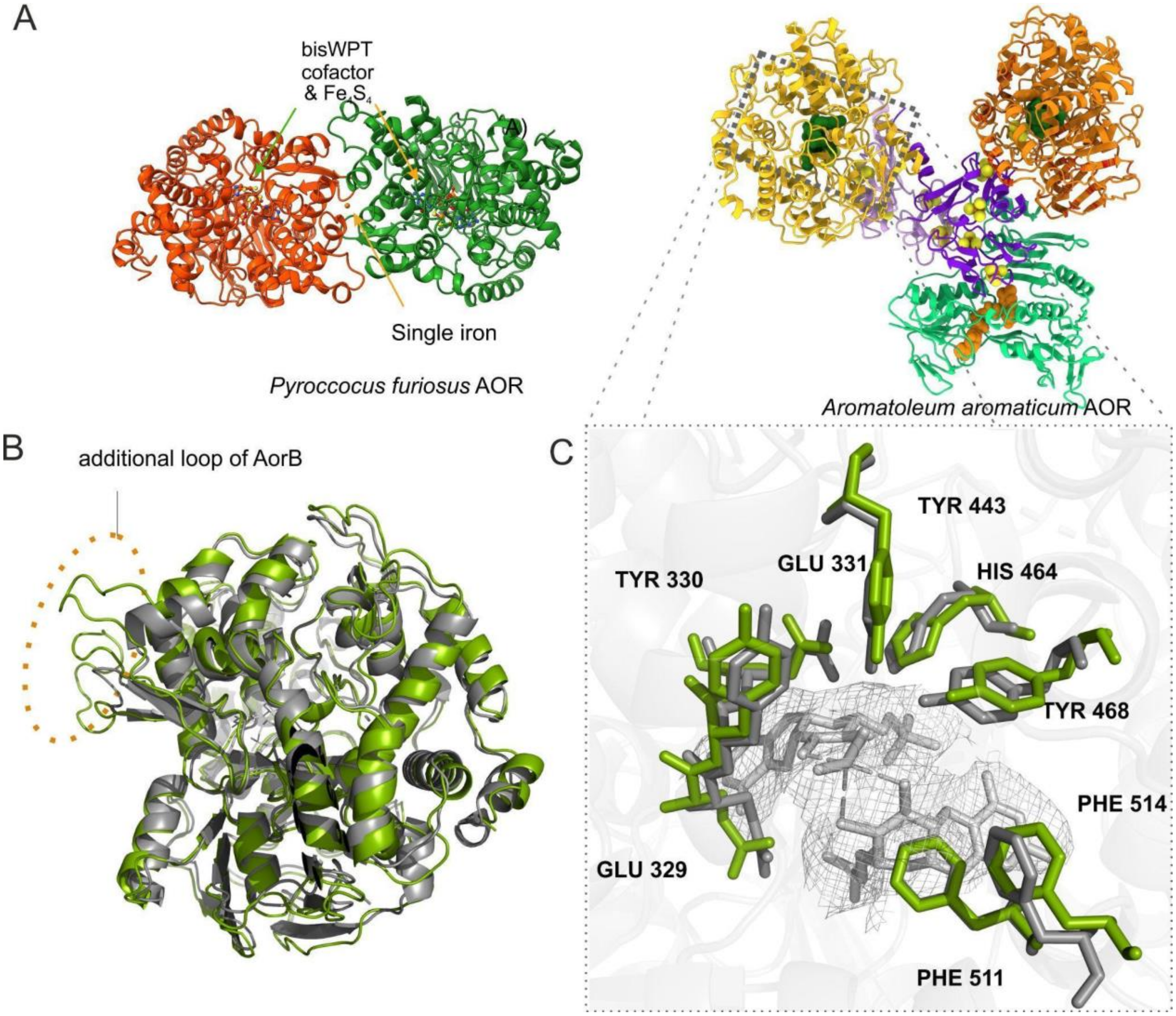

Fig. S5. Comparison between the structures of AOR_{Aa} and AOR from *P. furiosus* (AOR_{Pf}). A) Left: Structure of AOR_{Pf} (PDB: 1AOR); the iron atom as well as bisWPT cofactor and Fe_4S_4 cluster, are shown with arrows. Right: Structural model of AOR_{Aa}; B) Superposition of one AOR_{Pf} subunit (green) with AorB (grey) where an additional loop in AorB is shown (dashed circle); C) Zoom-in of the active site cavity of both superimposed AOR proteins (licorice). Residues numbering corresponds to AOR_{Aa}.

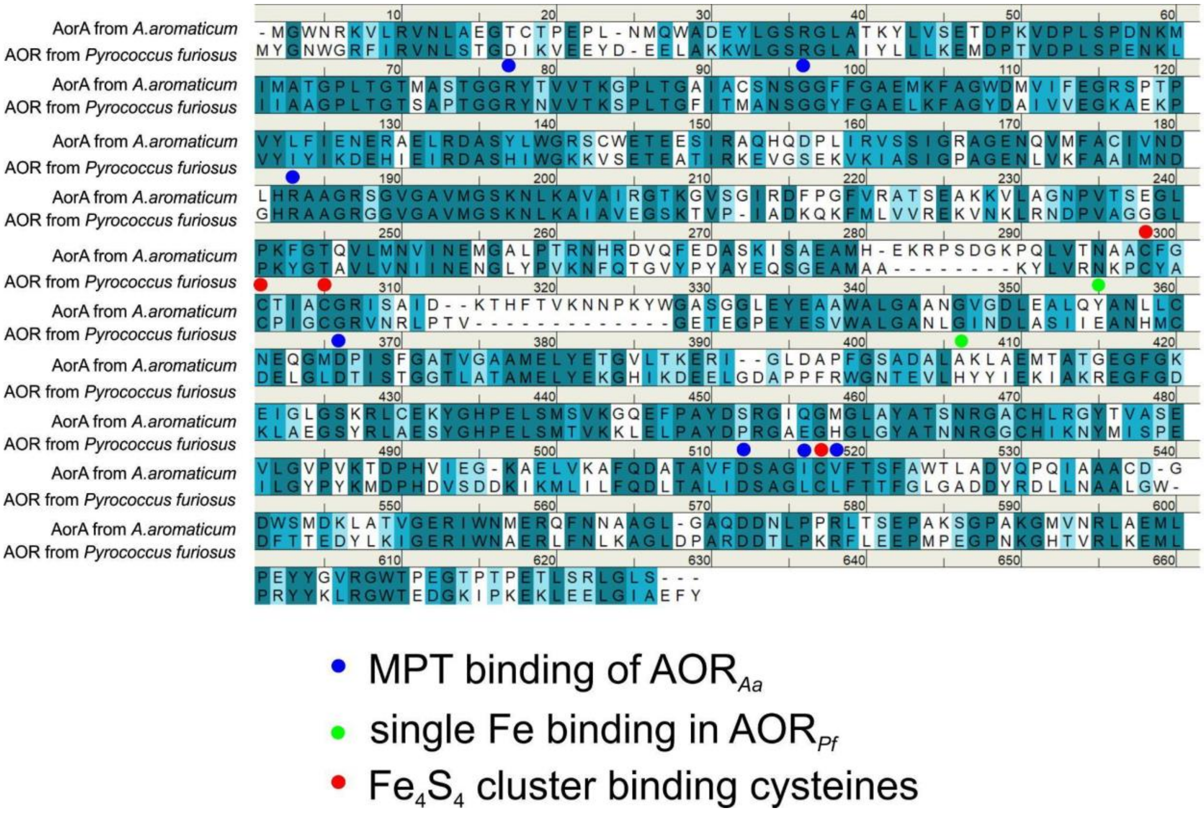

Fig. S6. Structural alignment of AorB and AOR from *P. furiosus* (PDB ID: 1AOR) sequences with marked residues responsible for binding the Fe_4S_4 cluster, MPT and a single Fe atom (present in 1AOR). Residues coloured in dark blue are identical, while those in lighter blue represent two degrees of similarity.

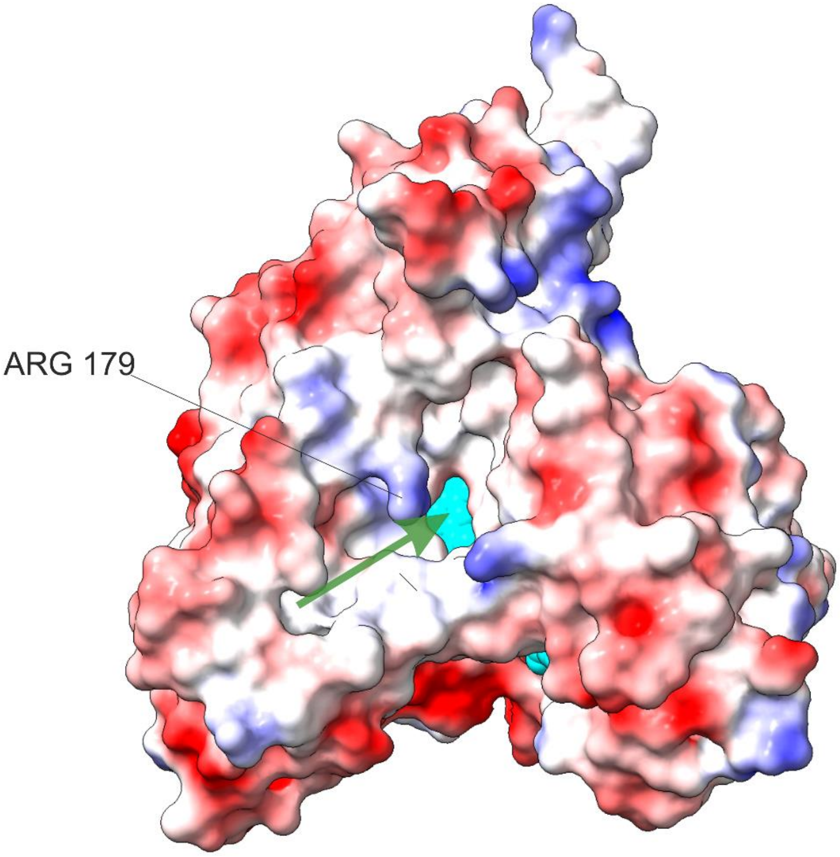

Fig. S7. FAD subunit surface coloured by electrostatic potential (blue: more positive; red: more negative). FAD is shown in cyan. An arrow marks the NADH binding pocket – positively charged – where the highly conserved Arg179 could facilitate the interaction.

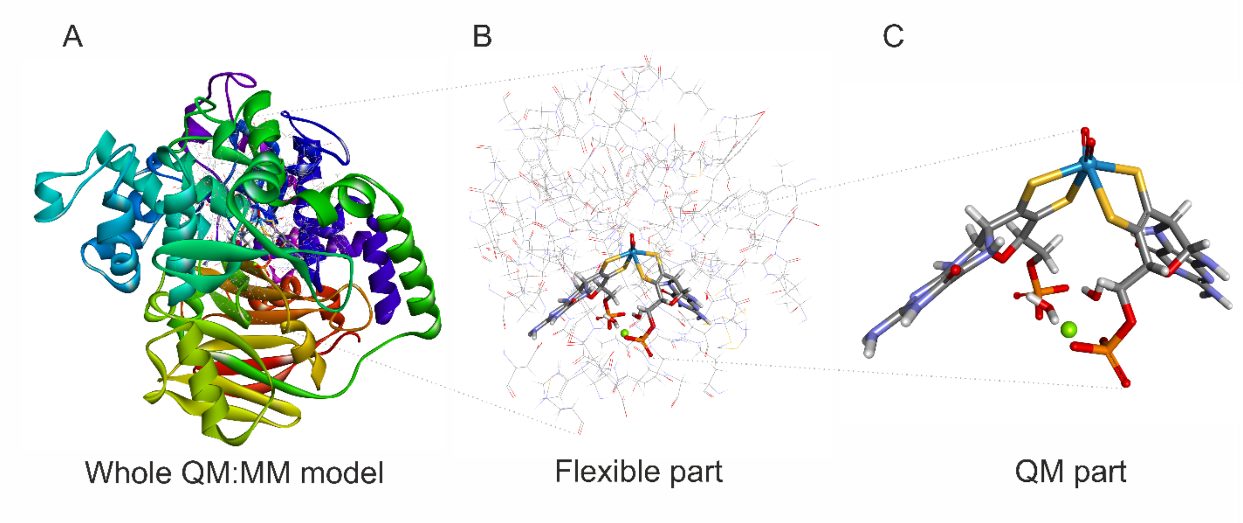

Fig. S8. QM:MM model of AOR$_{Pf}$ used for modelling W-co. A) Model of the complete AOR subunit; B) Flexible fraction of the AOR$_{Pf}$ model subjected to geometry optimization; C) QM:MM cofactor treated at a quantum chemical level.

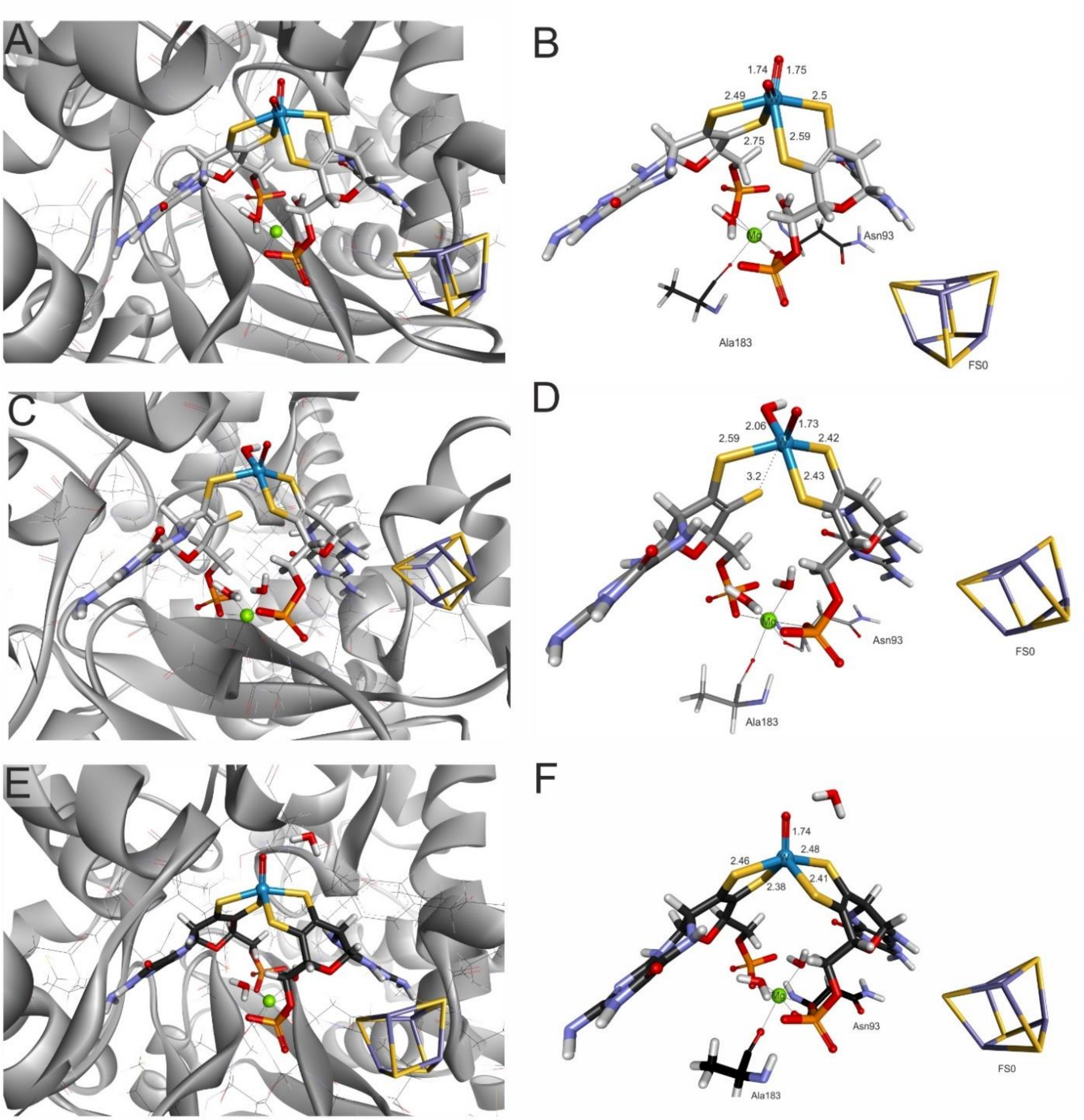

Fig. S9. QM:MM model of the active site in AOR from *P. furiosus*. W-co (sticks) surrounded by H-bond forming residues (lines) showing cofactor proximity to the Fe-S cluster FS0. A) and B) W(VI)O_2 - co-coordinated by two oxo ligands and two pterin moieties; C) and D) W(IV)O(OH) - co-coordinated by both an oxo and a hydroxo ligand, as well as a bidentate and monodentate pterin; E) and F) W(IV)O(OH_2) -co-coordinated by one oxo ligand and two pterin moieties; The Mg^{2+} atom is coordinated by two molecules of water, two phosphates and the carbonyl groups from Asn93 and Ala183.

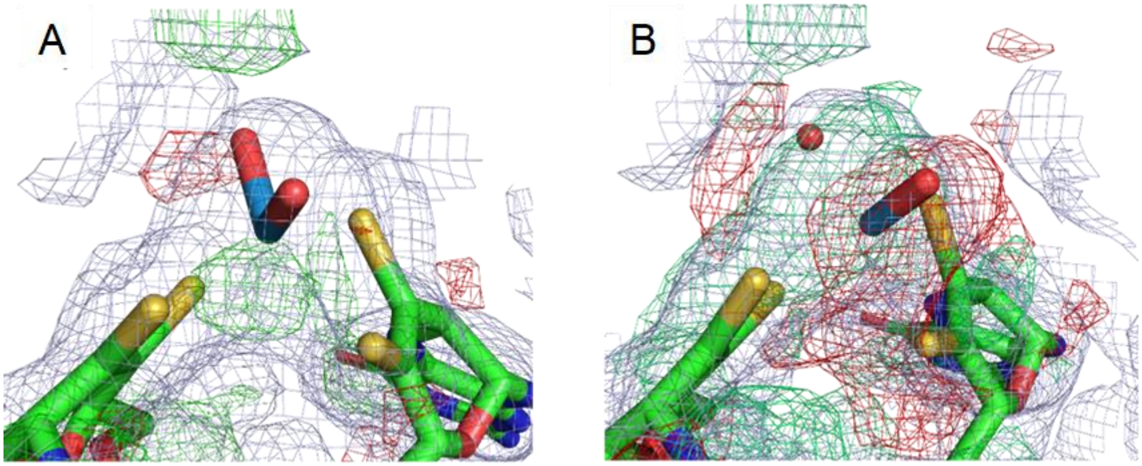

Fig. S10. Difference density maps calculated from AOR_{Pf} crystallographic data (density map corresponding to PDB ID: 1AOR) and different W-co versions modelled by QM:MM. A) Oxidized W(VI)OO model; B) Reduced W(IV)O(OH) model. For the processing with Phenix, the cofactor geometry was locked (rigid body fit) and tungsten occupancy was refined. The excess electron density of the model is shown in red, and the insufficient electron density of the model is shown in green.

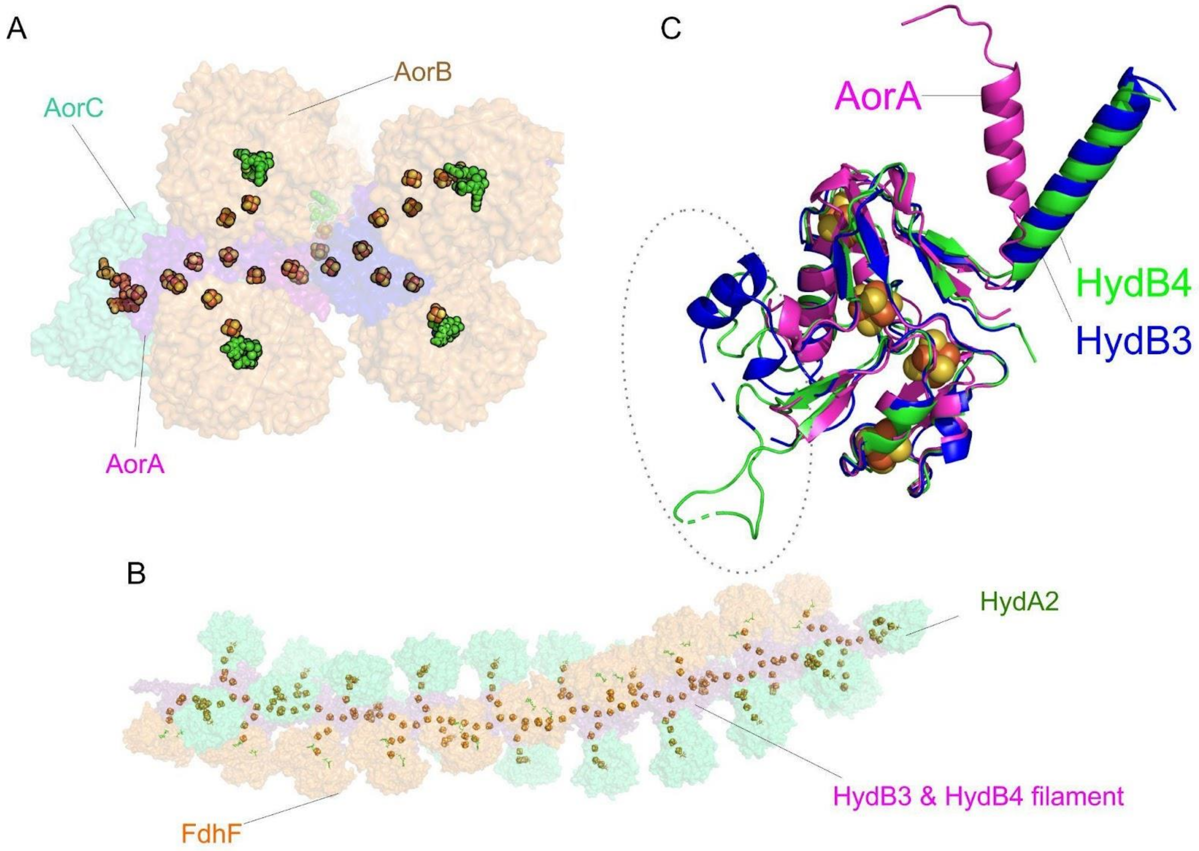

Fig. S11. Modelled nanowire composed by AOR$_{Aa}$ subunits. A) Modelled AOR complex of composition Aor(AB)$_5$C; B) Filament formation from independent AOR subunits; C) Structural alignment between the electron carrier cores of AOR$_{Aa}$ (composed by AorA subunits, magenta) and HDCR from *T. kivui* (PDB ID: 7QV7; composed by HycB3 and HycB4 in blue and green, respectively). FeS clusters are depicted as spheres.

Table S2. Activities of *Δhelix*-AOR in comparison to wild type AOR_{Aa}. The error was calculated by standard deviation.

Activity test	Specific activity of *Δhelix*-AOR [U/mg protein]	Specific activity of wild-type AOR_{Aa} [U/mg protein]
Benzaldehyde oxidation with BV^{2+} [a]	9.4 ± 0.6	23.4 ± 2.3
NAD^+ reduction with hydrogen[b]	0.68 ± 0.09	1.70 ± 0.09
Benzoic acid reduction with hydrogen[c]	0.24 ± 0.01	0.22 ± 0.01

[a] Activity test was conducted anaerobically (100% N_2 atmosphere) with 1 mM BV^{2+} as an electron acceptor, 0.4 mM benzaldehyde and 20 µg/mL of protein in 25 mM HEPPS buffer pH 8.0.

[b] Activity test was conducted anaerobically (97.5% N_2 and 2.5% H_2 atmosphere); hydrogen was the electron donor, 1 mM NAD^+ was reduced by 10 µg/mL of protein in 50 mM HEPES buffer pH 8.0, as described before[18].

[c] Activity test was conducted anaerobically (97.5% N_2 and 2.5% H_2 atmosphere) with 20 mM sodium benzoate and 20µg/mL of protein in 100 mM citric buffer pH 5.5, 0.5 mM NADPH and 170 µg/mL benzyl alcohol dehydrogenase, i.e., coupled assay as described before[18].

Other Supplementary Materials for this manuscript:

Data S1. (separate file)

QM:MM model of a single subunit of AOR_{Pf} with W(VI) coordinated by two oxo ligands and two pterins. Described in text as W(VI)OO model.

Data S2. (separate file)

QM:MM model a single subunit of AOR_{Pf} with W(IV) coordinated by oxo and hydroxo ligands and two pterins. Described in text as W(VI)O(OH) model.

Data S3. (separate file)

QM:MM model a single subunit of AOR_{Pf} with W(IV) coordinated by oxo and water ligands and two pterins. Described in text as W(IV)O(OH_2) model.

Data S4. (separate file)

Parameters for Fe_4S_4 cofactor used for MM calculations.

Data S5. (separate file)

Parameters for W-co cofactor used for MM calculations.

Data S6. (separate file)

Geometry of Fe_4S_4 cofactor used for MM calculations.

Data S7-S9. (separate file)

Geometry of parts of W-co cofactor used for MM calculations.
